# Evaluation of Production Protocols for the Generation of NY-ESO-1-Specific T Cells

**DOI:** 10.3390/cells10010152

**Published:** 2021-01-14

**Authors:** Wenjie Gong, Lei Wang, Sophia Stock, Ming Ni, Maria-Luisa Schubert, Brigitte Neuber, Christian Kleist, Angela Hückelhoven-Krauss, Depei Wu, Carsten Müller-Tidow, Anita Schmitt, Hiroshi Shiku, Michael Schmitt, Leopold Sellner

**Affiliations:** 1 Department of Internal Medicine V, Heidelberg University Hospital, 69120 Heidelberg, Germany; wenjie.gong@med.uni-heidelberg.de (W.G.); wang.lei@med.uni-heidelberg.de (L.W.); sophia.stock@hotmail.de (S.S.); ming.ni@med.uni-heidelberg.de (M.N.); maria-luisa.schubert@med.uni-heidelberg.de (M.-L.S.); brigitte.neuber@med.uni-heidelberg.de (B.N.); angela.hueckelhoven-krauss@med.uni-heidelberg.de (A.H.-K.); carsten.mueller-tidow@med.uni-heidelberg.de (C.M.-T.); anita.schmitt@med.uni-heidelberg.de (A.S.); michael.schmitt@med.uni-heidelberg.de (M.S.); 2 Department of Hematology, First Affiliated Hospital of Soochow University, Suzhou 215006, China; wudepei@suda.edu.cn; 3 Department of Hematology, The Affiliated Hospital of Guizhou Medical University, Guiyang 550025, China; 4 Department of Nuclear Medicine, Heidelberg University Hospital, 69120 Heidelberg, Germany; christian.kleist@med.uni-heidelberg.de; 5 National Center for Tumor Diseases (NCT), German Cancer Consortium (DKTK), 69120 Heidelberg, Germany; 6 Department of Immuno-Gene Therapy, Mie University, Tsu 514-8507, Japan; shiku@clin.medic.mie-u.ac.jp; 7 Takeda Pharma Vertrieb GmbH & Co. KG, 10117 Berlin, Germany

**Keywords:** NY-ESO-1-specific T cells, cell production protocols, adoptive cell therapy

## Abstract

NY-ESO-1-specific T cells have shown promising activity in the treatment of soft tissue sarcoma (STS). However, standardized protocols for their generation are limited. Particularly, cost-effectiveness considerations of cell production protocols are of importance for conducting clinical studies. In this study, two different NY-ESO-1-specific T cell production protocols were compared. Major differences between protocols 1 and 2 include culture medium, interleukin-2 and retronectin concentrations, T cell activation strategy, and the transduction process. NY-ESO-1-specific T cells generated according to the two protocols were investigated for differences in cell viability, transduction efficiency, T cell expansion, immunophenotype as well as functionality. NY-ESO-1-specific T cells showed similar viability and transduction efficiency between both protocols. Protocol 1 generated higher absolute numbers of NY-ESO-1-specific T cells. However, there was no difference in absolute numbers of NY-ESO-1-specific T cell subsets with less-differentiated phenotypes accounting for efficient in vivo expansion and engraftment. Furthermore, cells generated according to protocol 1 displayed higher capacity of TNF-α generation, but lower cytotoxic capacities. Overall, both protocols provided functional NY-ESO-1-specific T cells. However, compared to protocol 1, protocol 2 is advantageous in terms of cost-effectiveness. Cell production protocols should be designed diligently to achieve a cost-effective cellular product for further clinical evaluation.

## 1. Introduction

Adoptive cell therapy (ACT) with genetically engineered T cells expressing cancer-specific T cell receptors (TCR) is a promising cancer immunotherapy approach. These recombinant TCRs can recognize surface as well as intracellular tumor antigens. The cancer testis antigen New York esophageal squamous cell carcinoma-1 (NY-ESO-1) is an ideal immunotherapy target because NY-ESO-1 is widely expressed on a variety of cancer entities including melanoma [1,2], multiple myeloma [3] and soft-tissue sarcoma (STS) [2,4]. Importantly, NY-ESO-1 is only expressed on germinal tissue in adults [5,6]. Furthermore, NY-ESO-1 is an immunogenic protein as spontaneous humoral and cellular immune responses can be observed in cancer patients [7]. D’Angelo et al. demonstrated that engineered autologous T cells expressing NY-ESO-1c259 can mediate clinically meaningful antitumor effects in patients with metastatic synovial sarcoma [8]. Currently, numerous clinical trials using different NY-ESO-1-specific T cell products are initiated [9]. All of these studies are using different production protocols of genetically modified T cells. The manufacturing process can have an important impact on the efficacy of ACT [10]. Therefore, it is of great importance to evaluate different protocols for clinical application.

Ideal tumor-specific T cell products should successfully expand in vivo, achieve long-term persistence and, importantly, efficiently eliminate cancer cells. For this purpose, genetically modified T cells with high viability as well as transduction efficiency are required. In addition, the composition of the final cell product can influence the therapeutic success of ACT: For example, less differentiated T cells such as naïve-like (T_N_) or stem cell memory-like (T_SCM_) T cells have been reported to promote engraftment, long-term persistence, and extended tumor control [11,12].

In this study, we evaluated two different “ready-to-use” manufacturing protocols using retroviral vectors to generate NY-ESO-1-specific T cells. Activation and transduction strategies as well as the interleukin-2 (IL-2) concentrations used for T cell culture were major differences in the protocols applied. Importantly, these factors not only resulted in different production efficiencies and cellular product compositions, but also strongly affected the overall costs of the T cell production process.

## 2. Materials and Methods

### 2.1. Primary Cells

Peripheral blood (PB) samples from buffy coat of healthy donors (HDs) were obtained at the Heidelberg University Hospital. A ficoll density gradient (Linaris, Dossenheim, Germany) was performed to purify mononuclear cells which were cryopreserved in liquid nitrogen until usage. The study was approved by the Ethics Committee of the University of Heidelberg (S-254/2016). Informed consent was obtained from all HDs at the Blood Bank Heidelberg.

### 2.2. Cell Lines

The STS cell lines SW982 (NY-ESO-1+ HLA-A2+) and SYO-1 (NY-ESO-1- HLA-A2-) were kindly provided by Hiroshi Shiku (Mie University, Tsu, Japan). SW982 and SYO-1 cells were expanded in DMEM (Thermo Fisher Scientific, Waltham, MA, USA) supplemented with 10% heat-inactivated fetal bovine serum (FBS) (Thermo Fisher Scientific, Waltham, MA, USA).

### 2.3. NY-ESO-1-Specific T Cells Generation

**Figure 1:**
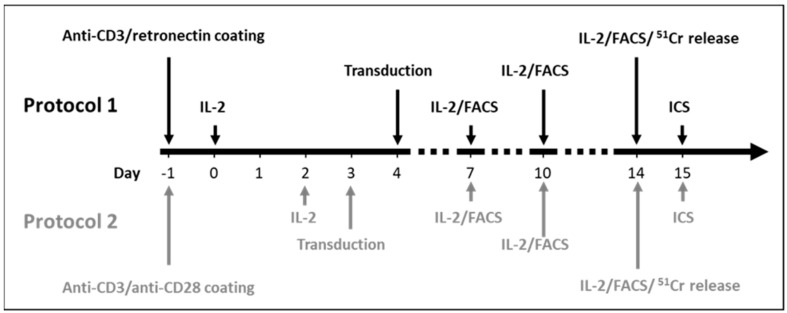
Time schedule until day 15 of the two protocols for NY-ESO-1-specific T cell generation. Abbreviations: IL-2: interleukin-2, FACS: fluorescence activated cell sorting, ^51^Cr: Chromium-51. ICS: intracellular cytokine staining.

Cryopreserved human PB mononuclear cells (PBMCs) from 6 different HDs were thawed and activated according to the two protocols, respectively. Protocol 1 was based on a NY-ESO-1 generation protocol employed in a clinical trial in Japan (NCT02869217). Protocol 2 was adapted from our clinical CD19 chimeric antigen receptor (CAR) trial for the treatment of CD19-positive hematological malignancies (NCT03676504) [13] using a third-generation retroviral vector system [14,15,16,17]. PBMCs from each HD were used for generation of NY-ESO-1-specific T cells applying the two protocols in parallel. This allows a direct comparison of the same donor samples between the two protocols. The main differences between the two protocols are illustrated in Figure 1 and Table 1. In both protocols, the first day of T cells activation was defined as day 0.

Protocol 1: GT-T551 (Takara Bio, Shiga, Japan) supplemented with 0.6% human serum (ZenBio, Durham, NC, USA) and 2% human serum albumin (US biological, Swampscott, MA, USA) was used for cell culturing. On day −1, the plates were pre-coated with 5 μg/mL anti-CD3 (Biozol, Eching, Germany) plus 25 μg/mL retronectin (Takara Bio). On day 0, PBMCs from HDs were added to the pre-coated plates and the culture medium was supplemented with 600 U/mL IL-2 (Novartis Pharma, Basel, Switzerland). Transduction was performed on day 4 with retronectin (20 μg/mL, Takara Bio) paired with spinoculation (centrifugation at 2000× *g*, 32 °C for 2 h).

Protocol 2: 45% RPMI 1640 (Thermo Fisher Scientific) and 45% Click’s Medium (EHAA) (Irvine Scientific, Santa Ana, CA, USA), supplemented with 10% heat-inactivated FBS (Thermo Fisher Scientific) and 2 mM L-glutamine (Thermo Fisher Scientific) was used for cell culturing. On day −1, the plates were pre-coated with 1 μg/mL anti-CD3 plus 1 μg/mL anti-CD28 (Biozol). On day 0, PBMCs from HDs were added to the pre-coated plates. The culture medium was supplemented with 100 U/mL IL-2 on day 2 of T cell culturing. Transduction was performed on day 3 with retronectin (7 μg/m, Takara Bio) paired with a static condition (37 °C for 1 h without centrifugation).

For both protocols, culture medium was changed routinely on days 7, 10 and 14 with addition of fresh IL-2. Transduction was performed with the MS3II-NY-ESO-1-siTCR retroviral vector based on the Moloney murine leukemia virus. Detailed information on the two protocols is provided in Supplementary Figure S1.

### 2.4. Flow Cytometry for Surface Markers

Cells were evaluated by multiparametric flow cytometry. Near-IR Dead Cell Stain Kit (Thermo Fisher Scientific) was used to exclude dead cells. HLA-peptide monomers were kindly provided by Prof. H. Shiku (Mie University, Tsu, Japan). PE-conjugated HLA NY-ESO-1-tetramer were produced according to the protocol of the NIH Tetramer Core Facility (Emory University, Atlanta, GA, USA) and were used to detect NY-ESO-1-specific TCR expression. The following fluorochrome-conjugated antibodies were used for immunophenotyping of following surface markers: anti-CD3-V510 (AmCyan), anti-CD8-PerCP, anti-CD45RA-APC, anti- programmed cell death protein 1 (PD-1)-Alexa Fluor 488, anti-T cell immunoglobulin mucin-3 (TIM-3)-Brilliant Violet 421 and anti-CXCR3-Alexa Fluor 488 (all from Biolegend, San Diego, CA, USA), anti-CD4-Alexa Fluor 700, anti-CCR7-PE-Cy7, anti-CD62L-eFluor 450 and anti-CD3-eFlour 610 (all from eBioscience, San Diego, CA, USA). Data were acquired on an LSR II device (BD Biosciences) and analyzed using FlowJo software (TreeStar, Ashland, OR, USA). The gating strategies are illustrated in Supplementary Figure S2.

### 2.5. Intracellular Cytokine Staining

Intracellular cytokine staining was performed as reported previously [18]. In brief, T cells were co-cultured with SW982 cells (NY-ESO-1+ HLA-A2+) or SYO-1 cells (NY-ESO-1- HLA-A2-) for 6 h in 96-well U-bottom microplates (Greiner BioOne, Frickenhausen, Germany). The cytokine secretion inhibitor Brefeldin A (BFA) was added for intracellular cytokine retention. Cells were then stained with NEAR-IR and surface marker antibodies followed by fixation and permeabilization using the Foxp3 fix/perm buffer set (Miltenyi Biotec, Bergisch Gladbach, Germany). Finally, cells were stained with anti-interferon gamma (IFN-γ)-Alexa Fluor 488 (Biolegend) and anti-tumor necrosis factor (TNF)-BV421 (BD Biosciences, Franklin Lakes, NJ, USA) at room temperature for 30 min. The gating strategies are illustrated in Supplementary Figure S2.

### 2.6. Cytotoxicity Assay

The specific anti-tumor efficacy of NY-ESO-1-specific T cells was measured using a 12-h Chromium-51 (^51^Cr; Hartmann Analytic, Braunschweig, Germany) release assay. SW982 cells (NY-ESO-1+ HLA-A2+) served as target cells, whereas SYO-1 cells (NY-ESO-1- HLA-A2-) were used as negative control. Cell lines were labeled with ^51^Cr for 2 h and co-incubated with NY-ESO-1 specific T cells or non-transduced T cells (effector cells) for 12 h using effector to target cell (E:T) ratios of 10:1, 5:1, 2.5:1 and 1:1 (5:1 only for non-transduced T cells) in 96-well U-bottom microplates (Greiner Bio-One) at 37 °C and 5% CO2. Maximum release and spontaneous release were determined by incubating the target cells with 1% Triton X-100 (Merck KGaA, Darmstadt, Germany) and medium alone, respectively. In addition, 75 μL of culture supernatant was diluted in Ultima Gold liquid scintillation cocktail (PerkinElmer, Waltham, MA, USA) and measured on a γ-counter (PerkinElmer). All experiments were performed in triplicates. Specific lysis was calculated according to the following formula: % specific lysis = (^51^Cr release in the test well—Spontaneous ^51^Cr release)/(maximum ^51^Cr release—Spontaneous ^51^Cr release) × 100.

### 2.7. Statistical Analysis

Statistical analysis was performed using Excel (Microsoft, Redmond, WA, USA). *p*-values were calculated using the paired *t*-test. *p*-values < 0.05 were considered statistically significant. Graphs and tables were designed using Excel, PowerPoint (Microsoft), Origin 2020 (OriginLab, Northampton, MA, USA) and Prism 6 (GraphPad Software Inc., San Diego, CA, USA). If not otherwise mentioned, results are presented as mean ± standard deviation (SD).

## 3. Results

### 3.1. Viability, Transduction Efficiency, and Expansion

**Figure 2:**
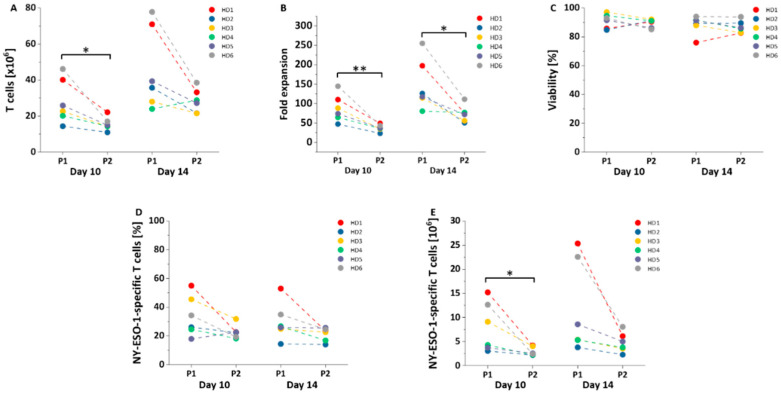
Viability, transduction efficiency and cell expansion. (**A**,**B**) Expansion of all T cells, (**C**) viability, (**D**) transduction efficiency and (**E**) NY-ESO-1 specific T cell expansion were assessed on day 10 and day 14 of the production (*n* = 6) using protocol 1 (P1) and protocol 2 (P2). Mean values were calculated for each group; error bars indicate standard deviation. Statistical significance was calculated with a paired two-way student *t*-test. Significance is represented as * for *p*-values < 0.05 and ** for *p*-values < 0.01.

Viability, transduction efficiency and expansion of T cells were determined longitudinally during the NY-ESO-1-specific T cell generation with PBMCs from six HDs. Cells were evaluated on days 10 and 14 of the T cell production process as harvesting for clinical application is usually performed in this time frame. Protocol 1 yielded significantly higher T cells on day 10 (P1 vs. P2: 28.21 ± 12.30 vs. 15.60 ± 3.73 × 10^6^ cells, *p* = 0.02, day 10; 45.99 ± 22.81 vs. 28.47 ± 6.65 × 10^6^ cells, *p* = 0.06, day 14; Figure 2A). The relative fold expansion was significantly higher on day 10 and day 14 applying protocol 1 (P1 vs. P2: 88 ± 37 vs. 37 ± 9, *p* = 0.007, day 10; 148 ± 67 vs. 74 ± 21, *p* = 0.02, day 14; Figure 2B). The viability (P1 vs. P2: 91 ± 5% vs. 89 ± 3%, *p* = 0.50, day 10; 88 ± 6% vs. 87 ± 4%, *p* = 0.47, day 14; Figure 2C) and transduction efficiency (P1 vs. P2: 34 ± 14% vs. 23 ± 5%, *p* = 0.08, day 10; 30 ± 13% vs. 21 ± 5%, *p* = 0.11, day 14; Figure 2D) of NY-ESO-1-specific T cells generated employing the two protocols were similar on day 10 and day 14. Protocol 1 generated more NY-ESO-1-specific T cells on day 10 (P1 vs. P2: 8.00 ± 5.13 vs. 2.93 ± 0.92 × 10^6^ cells, *p* = 0.04; Figure 2D). However, the differences of absolute numbers of NY-ESO-1-specific T cells did not reach statistical significance on day 14 (P1 vs. P2: 11.83 ± 9.57 vs. 4.79 ± 2.07 × 10^6^ cells, *p* = 0.08; Figure 2E).

### 3.2. Distribution of Different NY-ESO-1-Specific T Cells Subsets

**Figure 3:**
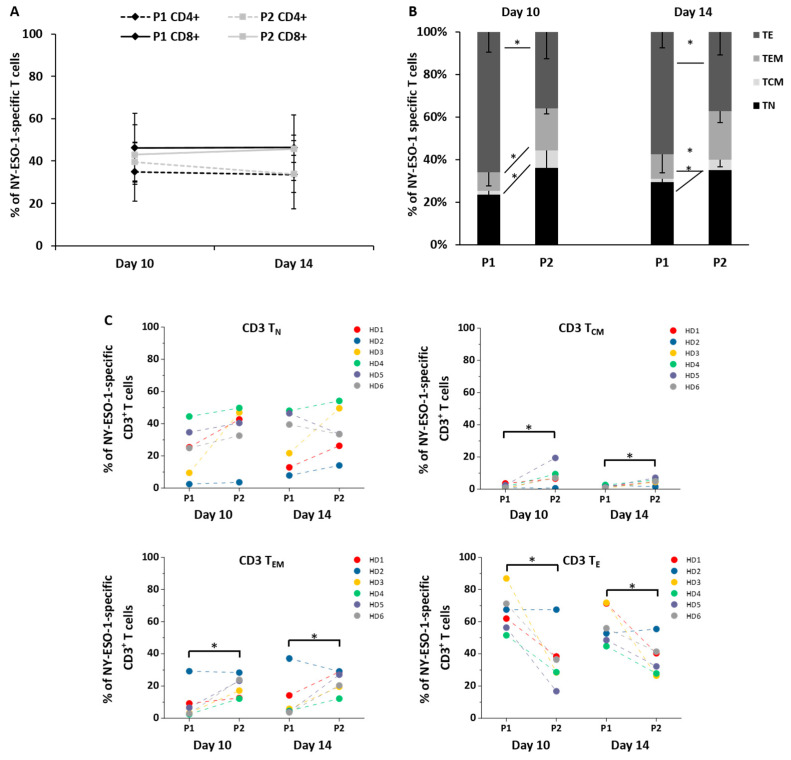
Distribution of different NY-ESO-1-specific T cell subsets. (**A**) The evolution of NY-ESO-1-specific CD4^+^ and CD8^+^ T cells (*n* = 6) was assessed on day 10 and day 14 of the production using protocol 1 (P1) and protocol 2 (P2); (**B**,**C**) comparison of the proportion of T_N_, T_CM_, T_EM_, and T_E_ cells among CD3^+^ NY-ESO-1-specific T cells (*n* = 6) on day 10 and day 14 between protocols 1 and 2. NY-ESO-1-specific T cells subsets were divided according to CD45RA and CCR7 expression. T_N_ cells were defined as CD45RA^+^CCR7^+^, T_CM_ cells as CD45RA^−^CCR7^+^, T_EM_ cells as CD45RA^−^CCR7^−^ and T_E_ cells as CD45RA^+^CCR7^−^ T cells. Mean values were calculated for each group; error bars indicate standard deviation. Statistical significance was calculated with a paired two-way student *t*-test. Significance is represented as * for *p*-values < 0.05.

The two protocols exerted a similar influence on the distribution of CD8^+^ and CD4^+^ NY-ESO-1-specific T cells in the cell product: On day 7, more CD4^+^ than CD8^+^ NY-ESO-1-specific T cells were the main subsets detectable within the T cell culture in both protocols. CD8^+^ NY-ESO-1-specific T cells progressively increased while CD4^+^ NY-ESO-1-specific T cells gradually decreased in the course of production and the ratio between CD8^+^ and CD4^+^ NY-ESO-1-specific T cells was reversed along with the culture duration regardless of the protocol used (Supplementary Figure S3). At the end of the production, comparable proportions of CD8^+^ NY-ESO-1-specific T cells as well as CD4^+^ NY-ESO-1-specific T cells for both protocols were observed (Figure 3A).

The NY-ESO-1-specific T cells were divided into four subsets according to CD45RA and CCR7 expression: naïve-like T (T_N_, CD45RA^+^CCR7^+^), central memory-like T (T_CM_, CD45RA^−^CCR7^+^), effector memory-like T (T_EM_, CD45RA^−^CCR7^−^), and terminally differentiated effector-like T (T_E_, CD45RA^+^CCR7^−^) cells. After in vitro activation, almost all T cells express CD95 [15]. Thus, T cells defined as T_N_ cells can be regarded as stem cell memory-like T (T_SCM_-like) cells in our study [19]. Protocol 1 rendered significantly higher proportions of T_E_ cells among all CD3^+^ NY-ESO-1-specific T cells on day 10 and day 14 (P1 vs. P2: 66 ± 13% vs. 36 ± 17%, *p* = 0.01, day 10; 57 ± 12% vs. 37 ± 11%, *p* = 0.03, day 14; Figure 3B,C), whereas significantly higher proportions of T_CM_ (P1 vs. P2: 2 ± 1% vs. 8 ± 6%, *p* = 0.04, day 10; 2 ± 1% vs. 5 ± 2%, *p* = 0.02, day 14; Figure 3B,C) and T_EM_ cells (P1 vs. P2: 9 ± 10% vs. 20 ± 7%, *p* = 0.03, day 10; 12 ± 13% vs. 23 ± 7%, *p* = 0.048, day 14; Figure 3B,C) were observed on day 10 and day 14 applying protocol 2.

### 3.3. Absolute Numbers of Different Subsets among NY-ESO-1-Specific T Cells

**Figure 4:**
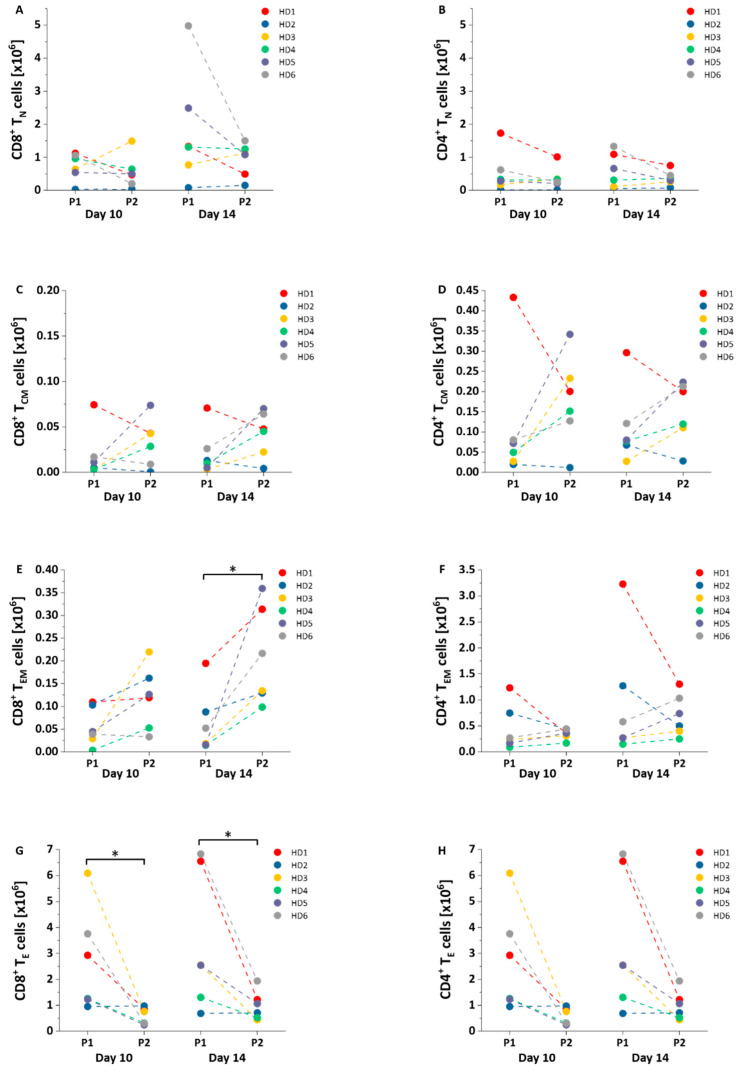
Absolute number of different NY-ESO-1-specific T cell subsets. (**A**–**H**) Differences in the absolute amounts of NY-ESO-1-specific T_N_ (**A**,**B**), T_CM_ (**C**,**D**), T_EM_ (**E**,**F**) and T_E_ (**G**,**H**) cells (*n* = 6) on day 10 and day 14 were compared between protocol (P1) and protocol 2 (P2). Mean values were calculated for each group; error bars indicate standard deviation. Statistical significance was calculated with a paired two-way student *t*-test. Significance is represented as * for *p*-values < 0.05.

Due to slower expansion (Figure 2D), protocol 2 resulted in lower absolute numbers of CD8^+^ T_N_ cells NY-ESO-1-specific T cells on day 14 compared to protocol 1, but this was without statistical significance (Figure 4A). No differences were observed in absolute numbers of CD4^+^ T_N_, CD8^+^ T_CM_, and CD4^+^ T_CM_ cells (Figure 4B–D). Protocol 2 increased the numbers of CD8^+^ T_EM_ cells on day 14 (P1 vs. P2: 0.06 ± 0.07 vs. 0.21 ± 0.11 × 10^6^ cells, *p* = 0.02, Figure 4E) rather than CD4^+^ T_EM_ cells (Figure 4F), while protocol 1 increased the amounts of CD8^+^ T_E_ cells (P1 vs. P2: 2.70 ± 2.00 vs. 0.58 ± 0.32 × 10^6^ cells, *p* = 0.046, day 10; 3.41 ± 2.64 vs. 0.98 ± 0.56 × 10^6^ cells, *p* = 0.043, day 14, Figure 4G) rather than CD4^+^ T_E_ cells (Figure 4H).

### 3.4. Exhaustion and Homing Markers

**Figure 5:**
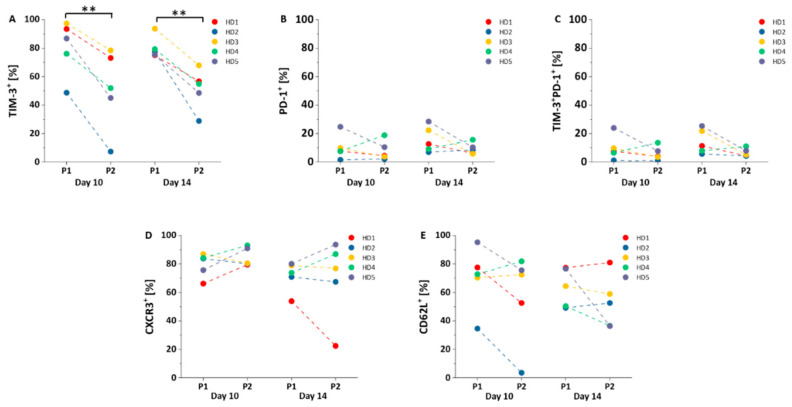
Exhaustion and homing markers of NY-ESO-1-specific T cells. (**A**–**C**) Comparison of TIM-3^+^ (**A**), PD-1^+^ (**B**), and TIM-3^+^PD-1^+^ (**C**) cells among NY-ESO-1-specific T cells (*n* = 5) between protocol 1 (P1) and protocol 2 (P2); (**D**,**E**) comparison of CXCR3^+^ (**D**) and CD62L^+^ (**E**) cells among NY-ESO-1-specific T cells (*n* = 5) between protocol 1 (P1) and protocol 2 (P2). Mean values were calculated for each group; error bars indicate standard deviation. Statistical significance was calculated with a paired two-way student *t*-test. Significance is represented as ** for *p*-values < 0.01.

The exhaustion markers T cell immunoglobulin mucin-3 (TIM-3) and programmed cell death protein 1 (PD-1), as well as the homing markers CD62L and CXCR3, were evaluated applying both protocols. Compared to protocol 1, protocol 2 significantly decreased the expression of TIM-3 (P1 vs. P2: 81 ± 20% vs. 51 ± 28%, *p* = 0.004, day 10; 80 ± 8% vs. 51 ± 14%, *p* = 0.005, day 14; Figure 5A). PD-1 levels were similar between the two protocols (Figure 5B). No significant differences were observed in the co-expression TIM-3^+^ PD-1^+^ subsets between the two protocols (Figure 5C). Furthermore, the proportions of CXCR3^+^ (Figure 5D) and CD62L^+^ cells (Figure 5E) among NY-ESO-1-specific T cells were similar between the two protocols.

### 3.5. Functional Evaluation of NY-ESO-1-Specific T Cells

^51^Cr release assay was performed on day 14 of T cells production. The STS cell line SW982 (NY-ESO-1^+^ HLA-A2^+^) was used as target cells. NY-ESO-1-specific T cells showed specific lysis of SW982 cells compared to NY-ESO-1^−^ HLA-A2^−^ SYO-1 cells (Supplementary Figure S4A). The lysis of SW82 cells by NY-ESO-1-specific T cells was significantly higher compared to non-transduced T cells with an E:T ratio of 5:1. Significantly more SW982 cells were lysed by NY-ESO-1-specific T cells generated with protocol 2 in comparison to protocol 1, especially at lower E:T ratios (P1 vs. P2: 18 ± 6% vs. 34 ± 3%, *p* = 0.01, E:T = 2.5:1; 5 ± 3% vs. 22 ± 6%, *p* = 0.02, E:T = 1:1; Figure 6A).

**Figure 6:**
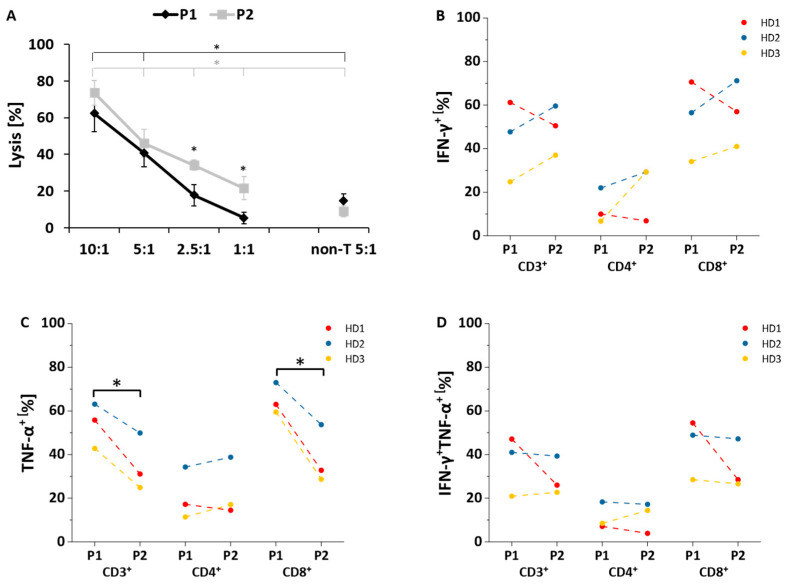
Functional evaluation of NY-ESO-1-specific T cells. (**A**) Cytotoxicity of NY-ESO-1-specific T cells cultivated with protocol 1 (P1) or protocol 2 (P2) was determined by ^51^Cr release assay after co-culture with NY-ESO-1^+^HLA-A2^+^ SW982 target cells for 12 h. Different effector (NY-ESO-1-specific T cells) to target (SW982 cells) ratios (10:1, 5:1, 2.5:1, 1:1) were used in technical triplicates to assess average lysis; (**B**–**D**) Intracellular production of IFN-γ and TNF-α in NY-ESO-1-specific T cells cultivated with protocol 1 (P1) or protocol 2 (P2) was determined after stimulation with SW982 cells for 6 h (*n* = 3). Overall, IFN-γ (**B**) and TNF-α (**C**) production as well as multifunctional NY-ESO-1-specific T cells producing both TNF-α and IFN-γ (**D**) were determined in CD3^+^, CD4^+^, and CD8^+^ T cells. Mean values were calculated for each group; error bars indicate standard deviation. Statistical significance was calculated with a paired two-way student *t*-test. Significance is represented as * for *p*-values < 0.05. non-T: non-transduced T cells generated under the same culture conditions.

Intracellular cytokine staining of IFN-γ and TNF-α was performed on day 15 of T cell generation. NY-ESO-1-specific T cells generated with both production protocols were incubated for 6 h with the STS cell line SW982 cells (NY-ESO-1^+^ HLA-A2^+^). There was no difference in IFN-γ levels in NY-ESO-1-specific T cells generated with the two production protocols (Figure 6B). In contrast, protocol 1 significantly upregulated TNF-α levels of all CD3^+^ (P1 vs. P2: 54 ± 10% vs. 35 ± 13%, *p* = 0.03) as well as CD8^+^ (P1 vs. P2: 65 ± 7% vs. 38 ± 13%, *p* = 0.02) but not of CD4^+^ NY-ESO-1-specific T cells (Figure 6C). Furthermore, the distribution of multifunctional NY-ESO-1-specific T cells capable of both IFN-γ and TNF-α production were assessed. There were no significant differences in the proportion of multifunctional NY-ESO-1-specific T cells generated with the two different production protocols (Figure 6D). No relevant baseline cytokine production of the NY-ESO-1-specific T cells was detectable with stimulation using NY-ESO-1^−^ HLA-A2^−^ SYO-1 cells or without stimulation (Supplementary Figure S4B,C).

## 4. Discussion

ACT with T cells recognizing the cancer testis antigen NY-ESO-1 is a promising treatment option for solid tumors such as STS. The ex vivo expansion and manipulation of NY-ESO-1-specific T cells is a crucial step for therapeutic success. However, various protocols differing in culture conditions including activation, transduction as well as supplemented cytokines are being used to generate these cells. By comparing two completely different manufacturing protocols, instead of elaborating a single aspect of a protocol, this study aims to provide the community “ready-to-use” manufacturing protocols for the generation of NY-ESO-1-specific T cells (and potentially ACTs with other targets). Both protocols can provide highly functional tumor-specific T cells and have individual advantages and disadvantages.

Both protocols generated NY-ESO-1-specific T cells with a high viability that reached approximately 90% during the production process. The T cell growth factor IL-2 is widely used for cultivation of T cells to maintain T cell proliferation and survival and serves as the basis of most adoptive T cell therapy protocols. IL-2 was supplemented to the culture medium in both production strategies, although the concentration differed dramatically: a higher concentration of IL-2 was used in protocol 1 (600 U/mL) compared to protocol 2 (100 U/mL) and might be a key parameter for the higher proliferation of T cell using protocol 1. Retrovirus-mediated gene transfer requires proliferating cells [20]. The higher proliferation rate observed applying protocol 1 may be, at least in part, responsible for the trend of the higher transduction efficiency and overall faster and superior yield of NY-ESO-1-specific T cells when compared to protocol 2. However, even the 6x lower concentration of IL-2 in protocol 2 was enough to provide highly functional, genetically modified T cells. Importantly, the high variability between different cell donor batches, especially using the higher IL-2 concentration, might have limited a clearer picture here.

Retronectin, the fragment of human fibronectin, enhances transduction efficiency by co-localizing viral vectors and the cells of interest, such as hematopoietic progenitor cells [21] and T lymphocytes [22]. Retronectin was used for transduction in both protocols. However, the concentration of retronectin and the binding strategy of retronectin with the virus differed: applying protocol 1, the retrovirus was pre-loaded on a higher concentration of retronectin (20 μg/mL) by 2 h centrifugation (spinoculation), before a second 10-min centrifugation of activated T cells was performed. In protocol 2, a lower concentration of retronectin (7μg/mL) was used to adsorb the retrovirus at 37 °C for 1 h (static), followed by a 5-min centrifugation of activated T cells. It was shown previously that, compared to static transduction, spinoculation could not only lead to a more than eightfold adsorption of retroviral vectors, but also increase the transduction efficiency by more than five fold [22]. Moreover, Chono et al. demonstrated that the gene transfer efficiency of lentiviral vectors was doubled after spinoculation when compared to a static pre-load [23]. Apart from retronectin and transduction strategy, efficient retroviral vector transduction depends on T cell division given that retroviral vectors can only integrate into the genome of proliferating cells. Protocol 1 led to a higher expansion of NY-ESO-1-specific T cells reaching significance on day 10. By making full use of high concentration of both retronectin and IL-2 applying protocol 1, a higher transduction efficiency was expected. However, in contrast to the above-mentioned studies, our data only showed a trend for increased transduction efficiency without reaching significance applying protocol 1. Nevertheless, the more labor- and cost-intensive protocol 1 could generate a significantly higher absolute number of NY-ESO-1-specific T cells.

A comparable proportion of CD8^+^ and CD4^+^ NY-ESO-1-specific T cells was generated with both protocols. Our observation that the duration of T cell culture can alter the balance between CD8^+^ cells and CD4^+^ cells towards CD8^+^ cells is in line with previous studies [15,24]. It was previously reported that anti-CD3/retronectin-based activation enriched for CD8^+^ T cells while anti-CD3/anti-CD28-based activation enriched for CD4^+^ T cells [25,26]. However, this effect was not observed in the current study at the relevant time points at day 10 and day 14 of the T cell production. In general, the role of CD4^+^ T cells in TCR-engineered T cells with MHC class 1 restrictions is not fully defined yet. 

When addressing the subsets of NY-ESO-1-specific T cells in detail, we observe that protocol 1 augmented the T_E_ cell subpopulation among NY-ESO-1-specific T cells. In contrast, protocol 2 enriched for T_CM_ and T_EM_ cells. No significant differences in the relative and absolute numbers of T_N_ cells between the two production protocols were observed. Accumulating evidence indicates that less-differentiated T cells such as T_N_ cells are important for longer persistence of engineered cells and sustained anti-tumor control in ACT [12,27,28,29]. The combination of anti-CD3/retronectin to activate T cells has been suggested to be superior for generating CD8^+^ T cells with a naïve-like phenotype compared to anti-CD3/anti-CD28-based activation [25]. This stands in contrast to the observation in our study. One of the explanations may be that the much higher IL-2 concentration used in protocol 1 compromised the effect of anti-CD3/retronectin-based activation. Kaartinen et al. evaluated various concentration of IL-2 ranging from 0 IU/mL to 300 IU/mL to generate CD19 CAR-T cells and reported that high IL-2 concentrations can decrease T_CM_ cells and augment T_E_ cells [24]. It is of note that, in contrast to previous findings in other ACT trials, no correlation of treatment response with the T cell phenotype in the applied cell product could be identified in a clinical trial evaluating NY-ESO-1-specific T cells in patients with synovial sarcoma [8]. However, the limited patient numbers in this study (*n* = 12) may limit clear conclusions and further analysis is necessary to evaluate the impact of less-differentiated T cells in NY-ESO-1-specific T cell products.

T cells of cancer patients undergo “exhaustion” as a deterioration of T cell function due to permanent exposure to tumor antigens [30]. Exhausted T cells display lower proliferation rates, less cytokine production, and lower cytotoxicity. The exhaustion is characterized by the overexpression of multiple inhibitory receptors such as TIM-3 and PD-1 [31]. TIM-3 expression on T cells is induced and maintained following chronic stimulation [32]. In our study, TIM-3 levels of NY-ESO-1-specific T cells were significantly higher under the conditions of protocol 1 when compared to protocol 2. This is in line with a previous study showing that TIM-3 expression can be increased by IL-2 in a dose-dependent manner [33]. In contrast, PD-1 levels of NY-ESO-1 specific T cells between the two protocols were similar. In particular, TIM-3^+^PD-1^+^ CD8^+^ T cells have been described to be in a more exhausted state compared to T cells expressing PD-1 alone [34,35]. In the current study, no significant differences in the proportion of TIM-3^+^PD-1^+^ cells were identified between the two protocols. Although exhausted T cells can be, in general, less effective in anti-tumor activity, it is unclear whether the sole higher expression of TIM-3 on these cells may have a negative effect on in vivo efficacy. Further evaluation is required to define the impact of TIM-3 and PD-1 expression on clinical outcome using NY-ESO-1-specific T cells.

The efficient homing of T cells and infiltration of the tumor tissue is critical in restraining tumor progression. CXCR3 is the predominant chemokine receptor mediating T_E_ cells recruitment to tumor tissue via its ligands CXCL9 and CXCL10, and has been demonstrated to be crucial for tumor control and survival [36,37]. Melanoma with low expression of ligands for CXCR3 is poorly infiltrated by T cells [38]. Moreover, CD62L has been reported to be a critical component for homing of T cells to the lymph node during initial homeostatic proliferation [39]. In the current study, we observed similar proportions of CXCR3^+^ cells and CD62L^+^ cells among the NY-ESO-1-specific T cells using the two protocols. Both homing markers were expressed on more than half of the NY-ESO-1-specific cells, and efficient homing into tumor tissue and lymph nodes can be expected using both protocols. 

NY-ESO-1-specific T cells generated by the two protocols displayed specific lysis of NY-ESO-1^+^ HLA-A2^+^ SW982 cells. However, the lytic capacity of NY-ESO-1 specific T cells generated by applying protocol 2 was superior compared to T cells employing protocol 1. The reasons for this difference in lytic capacity is unclear and is unexpected when taking into consideration the higher enrichment of T_E_ cells generated using protocol 1. However, the higher expression of TIM-3 in T cells generated according to this protocol may be involved in this finding.

CD8^+^ NY-ESO-1-specific T generated using protocol 1 displayed a significantly higher TNF-α level. This might partially be related to the higher percentage of T_E_ cells among NY-ESO-1-specific T cells using protocol 1. Similar percentages of IFN-γ^+^ NY-ESO-1-specific T cells and multifunctional NY-ESO-1-specific T cells producing both IFN-γ and TNF-α between both protocols were observed. This raises the question of why NY-ESO-1-specific T cells produced by protocol 1 secreted more TNF-α but had lower lytic activity when compared to cells generated using protocol 2. According to Chao Ma et al., polyfunctional T cells are highly functional T cells that dominate the anti-tumor immune response. These cells can secrete 100 times more copies of a given protein than non-polyfunctional T cells in spite of their relatively low proportion among all T cells [40]. In addition, a previous study in mouse models has demonstrated that the overall quality of an immune response is best reflected by the effector function of polyfunctional T cells [41]. Therefore, the secretion of a single cytokine is probably not sufficient to assess anti-tumor efficacy. The discrepancy between lysis and TNF-α secretion in our study implied the role of multifunction T cells. Furthermore, other factors, e.g., the higher expression of exhaustion markers such as TIM-3 in NY-ESO-1-specific T cells generated with protocol 1 may be accountable for the differences in cytotoxicity. Importantly, ex vivo lysis and cytokine production assays are crucial to determine general functionality of genetically modified T cells, but the results cannot directly reflect their efficacy in clinical application.

Finally, it is important to highlight that, in contrast to protocol 1, protocol 2 is relying on FBS for T cell cultivation. We are using FBS for the generation of GMP-grade CAR-T cells in our clinical trial (NCT03676504) but extensive washing steps at the end of the production process are necessary to reduce the amount of FBS in the final cell product. Furthermore, the use of FBS for clinical cell therapy application may be restricted in some countries. In these terms, the FBS-free protocol 1 may have advantages over protocol 2 in specific occasions where FBS has to be avoided for GMP-grade T cell cultivation.

In conclusion, rather than evaluating a single aspect of an ACT production protocol, we compared two “ready-to-use” protocols that can be directly adapted by the community. Both protocols can efficiently manufacture functional NY-ESO-1-specific T cells. However, both protocols endow advantages and disadvantages: Protocol 1 can achieve a faster T cell expansion with enrichment of CD8^+^ T_E_ NY-ESO-1-specific T cells. In contrast, protocol 2 can promote a higher number of less-differentiated T cells with a lower expression of exhaustion markers. This is particularly important, as the crucial problem in current therapies with genetically modified T cells is longevity of the T cell population and function. Here, transduced T_N_ cells are particularly helpful as they can later differentiate into effector T cells, thus ensuring a long-lasting action of genetically altered T cells. Protocol 1 as the more expensive and more labor-intensive protocol may be preferred if there is need to quickly generate the cellular product. Protocol 2 seems to be more cost-effective and may generate a lower number of NY-ESO-1-specific T cells that are potentially of higher quality and functionality. A higher proportion of NY-ESO-1-specific T cells with a more favorable phenotype may need a lower absolute number of cells transfused for efficient engraftment and tumor control. The transfer of a lower number of T cells may also be accompanied by a more favorable safety profile. In vivo studies will be necessary to confirm the comparability or even superiority of one of these protocols.

## Figures and Tables

**Table 1:** Major differences in the protocols for the generation of NY-ESO-1 specific T cells.

	Protocol 1 (P1)	Protocol 2 (P2)
**Culture Medium**	GT-T551	45% RPMI 1640
	2% human serum albumin	45% Click’s Medium (EHAA)
	0.6% human serum	10% heat-inactivated FBS
		2 mM L-glutamine
**Activation**	Anti-CD3 (5 μg/mL) +	Anti-CD3 (1 μg/mL) +
	RetroNectin (25 μg/mL)	Anti-CD28 (1 μg/mL)
**Cytokines**	600 U/mL IL-2	100 U/mL IL-2
**Retronectin used for transduction**	20 µg/mL	7 µg/mL
**Transduction**	1 mL retrovirus 2000 g 32 °C 2 h	1 mL retrovirus 37 °C 1 h
	(spinoculation)	(static)

## Supplementary Materials

The following are available online at https://www.mdpi.com/2073-4409/10/1/152/s1. Figure S1: Details of protocol 1 and protocol 2, Figure S2: Gating strategy from a representative production, Figure S3: The evolution of NY-ESO-1-specific CD4^+^ and CD8^+^ T cells on days 7 and 10 of the production using protocol 1 and 2, Figure S4: Specificity of NY-ESO-1-specific T cells for NY-ESO-1^+^HLA-A2^+^ cells.
