# Supplementary material for: Evaluation of Production Protocols for the Generation of NY-ESO-1-Specific T Cells
Source: Cells. 2021 Jan 14;10(1):152. doi: 10.3390/cells10010152 (PMC7828728; doi:10.3390/cells10010152)
Supplement: Supplementary file 1 [file cells-10-00152-s001.pdf]

Supplementary Figure 1.

	Protocol 1	Protocol 2
**Day -1: pre-coating**	Coat 12-well plate with 5 µg/ml anti-CD3 plus 25 µg/ml retronectin	Coat 24-well plate with 1 µg/ml anti-CD3 plus 1 µg/ml anti-CD28
	Incubate at 4°C overnight	
**Day 0: activation of PBMCs**	Aspirate the supernatant	
	Wash with GT-551 for 3 times	Add culture medium (CM), incubate at 37°C, 5% CO_2 for 30 min
	Aspirate the supernatant, thaw and add PBMCs	
	$5.2x10^5$ PBMCs/ well in 2.6 ml CM with IL-2 (600 U/ml)	$1x10^6$ PBMCs/ well in 2 ml CM without IL-2
**Day 2: adding IL-2 and pre-coating**		First feed with IL-2 (100 U/ml)<br>Coat 24-well plate with retronectin (7µg/ml), incubate at 4°C overnight
**Day 3: pre-coating and transduction**	Coat 24-well plates with retronectin (20 µg/ml), incubate at 4°C overnight	**Static condition:**<br>Aspirate supernatant<br>Washing with CM<br>Adding 500 µl/well retroviral supernatant, incubate at 37℃ 5% CO for 30min<br>Repeat above once again<br>Add $5x10^5$ activated T cells (ATCs)/ well in 2 ml CM with IL-2, centrifuge 1000 g for 5 min at RT, incubate at 37°C, 5% CO_2 until D7
**Day 4: transduction**	**Spinoculation:**<br>Aspirate supernatant<br>Wash with ACD-A twice<br>Add 1 ml/well of retroviral supernatant, centrifuge at 2000 g 32°C for 2h (accelerate fast, brake slow)<br>Aspirate retroviral supernatant<br>Wash with PBS containing 1.5% human serum albumin (HSA)<br>Add 3.8 $x10^5$ ATCs/ well in 0.98 ml CM with IL-2, centrifuge 1000 g for 10 min at 32°C, incubate at 37°C, 5% CO_2	
**Day 5: transferring**	Transfer the cells into T25 flask, incubate at 37°C, 5% CO_2 until D7	
**Day 7, Day 10, Day 14**	Expansion with new addition of IL-2, FACS for immunophenotype assessment	
**Day 14**	Chromium-51 (^{51}Cr) release assay	
**Day 15**	Intracellular cytokine staining (ICS)	

Supplementary Figure 1. Details of protocol 1 and protocol 2

The details of the two protocols are displayed chronologically. NY-ESO-1-specific T cells were generated according to the above procedures, respectively. Abbreviations: CM: culture medium, ATCs: activated T cells, HAS: human serum albumin, ^{51}Cr: Chromium-51, ICS: Intracellular cytokine staining.

Supplementary Figure 2

A.

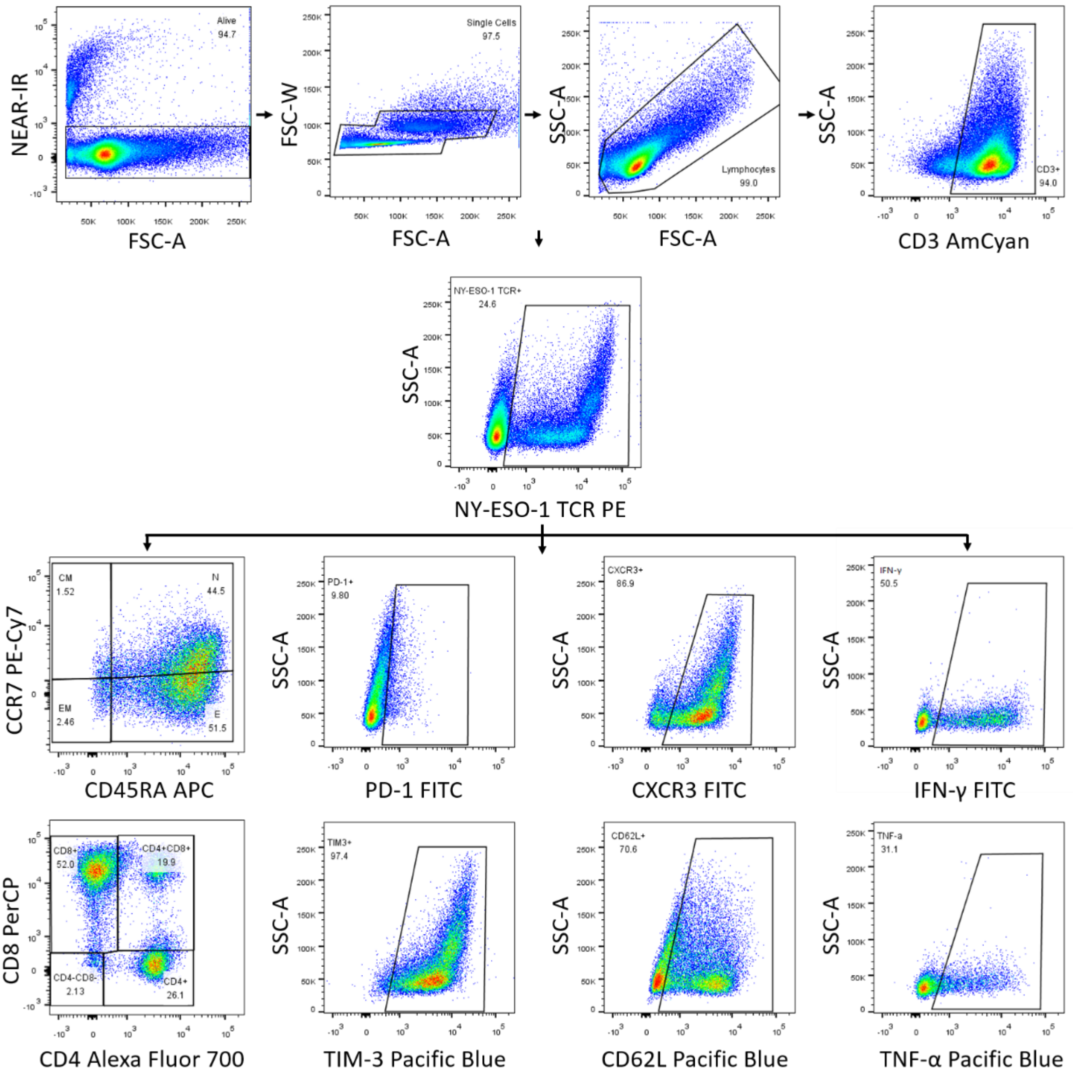

B.

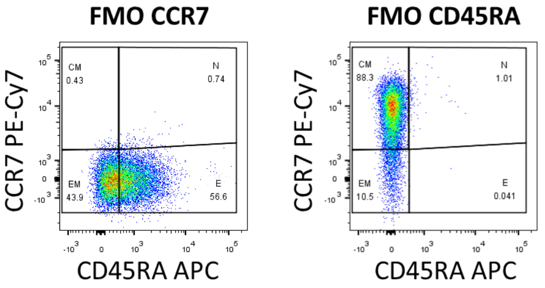

C.

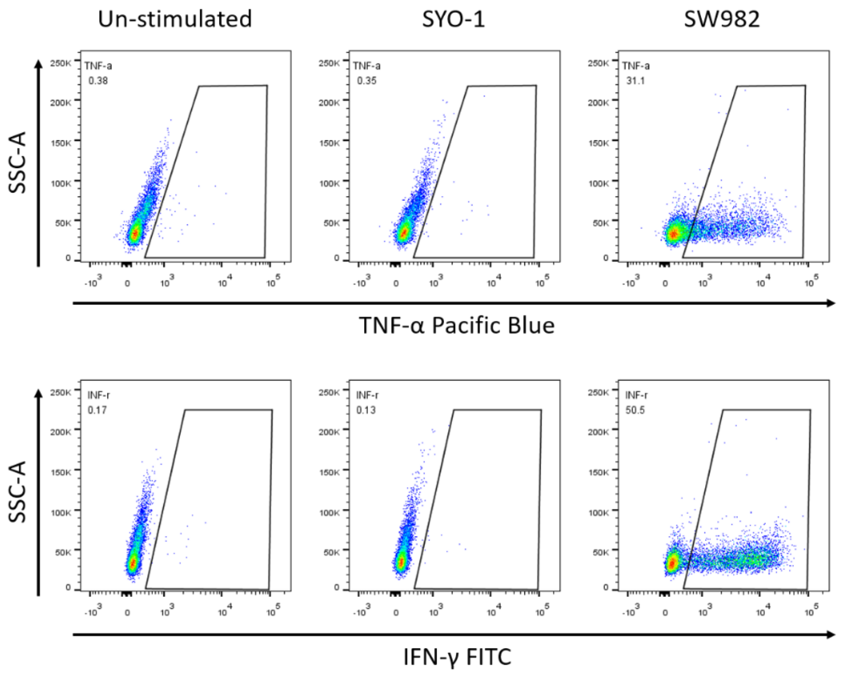

Supplementary Figure 2. Gating strategy from a representative production.

(**A**) NY-ESO-1-specific T cells from a representative healthy donor (HD) on day 10 and day 15 were analyzed. (**B**) Naïve-like T (T_N) cells were defined as CD45RA$^+$CCR7$^+$, central memory-like T (T_{CM}) cells as CD45RA$^-$CCR7$^+$, effector memory-like T (T_{EM}) cells as CD45RA$^-$CCR7$^-$ and effector-like T (T_E) cells as CD45RA$^+$CCR7$^-$ T cells. Fluorescence minus one (FMO) controls of CD45RA and CCR7 were used to define the gates. (**C**) Representative intracellular cytokine staining plots for TNF-α and IFN-γ. Unstimulated, as well as SYO-1 (NY-ESO-1$^-$ HLA-A2$^-$) and SW982 (NY-ESO-1$^+$ HLA-A2$^+$) stimulated cells are shown.

Supplementary Figure 3.

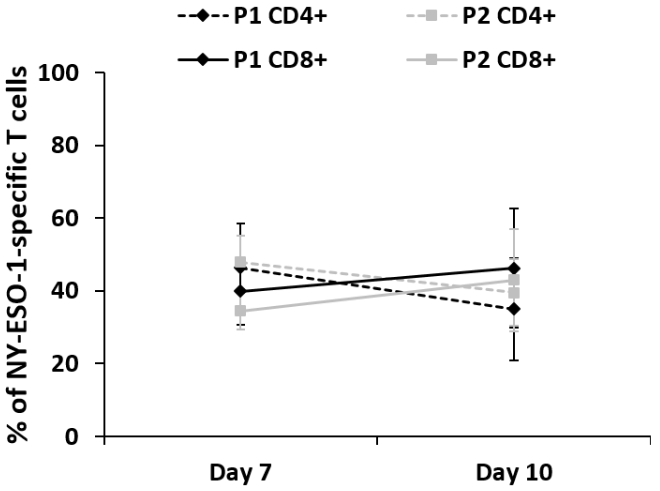

Supplementary Figure 3. The evolution of NY-ESO-1-specific $CD4^+$ and $CD8^+$ T cells on days 7 and 10 of the production using protocol 1 and 2.

On day 7 of T cell production, the perctentage of NY-ESO-1-specific $CD4^+$ T cells was 46% while that of NY-ESO-1-specific $CD8^+$ T cells was 40% applying protocol 1 (P1). The perctentage of NY-ESO-1-specific $CD4^+$ T cells was 48% while that of NY-ESO-1-specific $CD8^+$ T cells was 34% applying protocol 2 (P2).

Supplementary Figure 4.

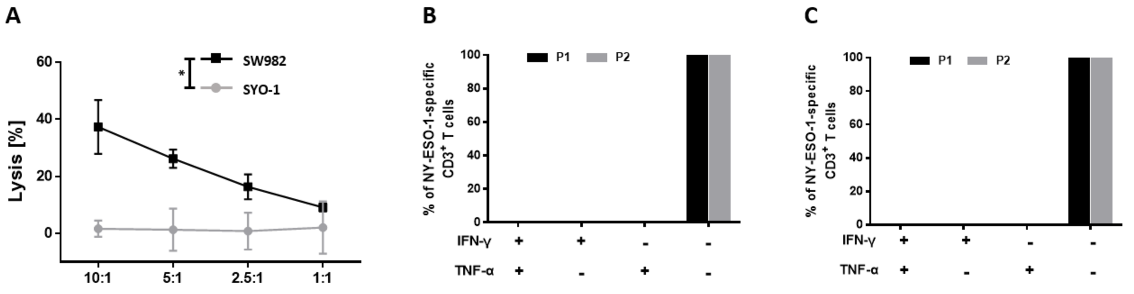

Supplementary Figure 4. Specificity of NY-ESO-1-specific T cells for NY-ESO-1^{+}HLA-A2^{+} cells.

(**A**) ^{51}Cr release assay was used to determine the cytotoxicity of fresh NY-ESO-1-specific T cells after co-culture with the STS cell lines SW982 cells (NY-ESO-1^{+}HLA-A2^{+}) and SYO-1 cells (NY-ESO-1^{-}HLA-A2^{-}) for 12 hours. Different effector (NY-ESO-1-specific T cells) to target (STS cell lines) cell ratios (10:1, 5:1, 2.5:1 and 1:1) were used. The cytotoxic lysis of SYO-1 cells (gray line) was significantly lower compared to that of SW982 cells (black line), demonstrating specific lysis of NY-ESO-1^{+} HLA-A2^{+} cells by NY-ESO-1-specific T cells. (**B-C**) Representative levels of TNF-α and IFN-γ in NY-ESO-1-specific T cells after co-incubation with SYO-1 cells (NY-ESO-1^{-}HLA-A2^{-}) for 6 hours (**B**) or without stimulation (**C**) are displayed. No remarkable activity of NY-ESO-1-specific T cells by stimulation with NY-ESO-1^{-}HLA-A2^{-} SYO-1 cells or without stimulation was observed. All experiments were performed in triplicates. Mean values were calculated for each group. Error bars indicate standard deviation. Statistical significance was calculated with the parametric two-way student t test. Significance is represented as * for p-values < 0.05.
